# Domestic Summary

**Published:** 1840-04-25

**Authors:** 


### DOMESTIC SUMMARY.

*Note upon Gentiana Chirayita.*—To the extensive and well known family of Gentianeæ, belong numerous species which are valuable for their medicinal qualities. Although closely resembling each other in botanical characters, they are equally remarkable for the similarity of property, connected with their bitterness, which universally pervades them, and which, with few exceptions, permits the substitution of one for another, when employed as medicines. In the Flora of every explored region of the earth, are found one or more individuals which have been ascertained to possess the qualities of the class in an eminent degree, and on this account have been selected to occupy a place in the list of the Materia Medica peculiar to that region. The species under consideration is a native of India, whence it has been brought to Europe, and, within a few years, has attracted some attention. The information we possess of its history and virtues is derived from several sources; upon these we draw for the remarks to be presented to our readers.

The following are the names given to it by different authors:

*Gentiana chirayita.*—Roxb. *Hor. Corom. and Asiat. Researches.*

*Henricea pharmacearcha.*—Lem. Lis. *Bul. Soc. Philom.*

*Swertia chirayita.*—Hamilton.

*Description.*—This plant is herbaceous, two or three feet high, branched; the stems are woody, as thick as straws, round, smooth, and jointed, containing a large medullary canal, of a yellow colour; the leaves are amplexicaul, lanceolate, acute, entire, smooth, and three or five-veined; the flowers are yellow, in terminal spikes; the corolla is five-parted.

It has no odour, and the taste is very bitter. In the Linnæan arrangement it belongs to the class *Pentandria*, order *Digynia.*

Chirayita is found upon the Coromandel coast of the Peninsula, and in the district of Nepal.

We are informed by Pereira that it is imported into England tied up in bundles, and that the plant is pulled up by the root, about the time the flowers begin to decay, and when the capsules are well formed. That which we have received is cut into small fragments.

Dr. Ainslie says, what appears in the bazaars of Lower India, under the Tamul name of *chayret toochie*, are small stalks, of a light gray colour, and very bitter but pleasant taste.

An analysis of the plant has been made by Lassaigne and Boissel, who present, as their results, the following composition: resin, yellow bitter matter, brownish-yellow colouring matter, gum, malic acid, chloride of potassium, sulphate of potassa, phosphate of lime, and oxide of iron.

In India it is employed as a stomachic in dyspeptic complaints, and as a febrifuge in intermittents. According to Roxburgh, it is prescribed as a substitute for cinchona, when that bark cannot be procured. The credit of making it known in Europe appears to be due to M. Leschenault. Besides the tonic power which, like all its congeners, it possesses to a considerable extent, others have been claimed for it, which, if verified by experience, will much enhance its value as a remedial agent; we fear, however, that partiality for a new substance has carried its advocates too far in their encomiums, as it exhibits too little difference of composition, when compared with other species of gentian, for the existence of marked difference in properties. Thus, Dr. Currie has supposed "that he recognised in it an especial action upon the abdominal organs, especially upon the liver, for, during its use, the stools became more bilious, the complexion clearer, and he was induced to employ it in obstructions." And in his lectures, published in 1838, Dr. Sigmond will be found to employ the following language: "It seems that not only does it act upon the stomach, imparting to it a greater degree of vigor, so that the increase of the gastric juice is attendant upon it, and thus the first process of digestion promoted, but the secretion of the liver is materially improved by it, for I have always found that, where it has been given, the stools have speedily acquired the healthy tinge of bile, and also the muscular activity of the bowels has been increased, for the peristaltic action becomes more regular, and performed with more decided periodicity." "Its beneficial effects are generally more permanent than the greater number of bitters, nor does it, as most of the barks, woods, and roots which we employ for dyspeptic states, and for all that host of morbid affections which depend upon disordered function of the stomach and bowels, ever constipate the bowels, or interfere with the healthy function of the liver; on the contrary, it corrects the secretion of the bile, and gently operates on the bowels." And again, "I have often found chirayita very much to be preferred to sarsaparilla, when large quantities of mercury have been taken, and often, after salivation has been produced, the system more quickly recovers its lost equilibrium than from the use of any other drug with which I am acquainted. It has likewise been strongly recommended in leucorrhœa, dependant upon a general relaxed condition of the female frame; it has even been called a specific remedy. At that period of life in which the menstrual secretion is about to disappear, and in which there is great carefulness to be remembered, lest the employment of medicines injudiciously may lay the foundation for disease of the uterus, or in the mammæ, this tonic is very effectual; it produces no determination to any of the organs, but combines the power of invigorating, with that of removing obstructions." Whether this is a high wrought picture of the effects to be derived from the therapeutic application of this new remedy, we leave to be determined by future observations.

Chirayita yields its virtues to water and alcohol. A concentrated infusion is productive of nauseating and irritating effects upon the stomach; that made of the strength of half an ounce of the plant to the pint of water, is sufficient for all purposes.

A formula is given by Dr. Sigmond, for the preparation of the tincture, which is, to macerate five ounces of the chirayita for fourteen days in two pints of proof spirit. "This contains all the powers of the herb; it forms a very strong but very pleasant bitter, by no means unpalatable. It is grateful to the stomach, and diffuses throughout the system a general warmth." The dose is a tea-spoonful.

If given in substance, the dose is one scruple, powdered.

Another point of interest connected with this plant arises from the circumstances of its having been supposed by Guibourt, to constitute the *Calamus verus* of the ancients. This supposition is based by him upon its characters. It has been shown, however, by Fee, by drawing a parallel between the description of the two plants, that such an assumption cannot be relied on, as the characters of *Calamus verus*, which are given by Theophratus, Dioscorides, and Pliny, have no correspondence with those of gentian, and the sensible qualities also are different. J. C.

*American Journal of Pharmacy.*

